# RxReduce: a medication deprescribing agent framework leveraging large language models to optimize proton pump inhibitor use

**DOI:** 10.1093/jamiaopen/ooag167

**Published:** 2026-10-09

**Authors:** Benjamin Michaels, Reed Evans, Kris Riedman, Joanna Yang, Gray Selby

**Affiliations:** Department of Pharmacy, UCSF Health, San Francisco, CA 94158, United States; Amgen Inc R&D, Thousand Oaks, CA 91320, United States; Microsoft, Redmond, WA 98052, United States; Mount Sinai Health System, New York, NY 10029, United States; The Gund Company, St Louis, MO 63132, United States

**Keywords:** polypharmacy, decision support systems, artificial intelligence, proton pump inhibitors, deprescribing

## Abstract

**Objectives:**

Polypharmacy rates continue to rise which can lead to increased risk of adverse drug events. Deprescribing is essential but time-consuming, requiring extensive clinical note and chart review. We developed RxReduce, a large language model (LLM)-powered framework as a proof of concept that can assist with medication deprescribing.

**Materials and Methods:**

RxReduce utilizes LLMs and retrieval-augmented generation (RAG) to evaluate patient data. Focusing on proton pump inhibitors, the system analyzes diagnoses and clinical notes to generate recommendations (Continue, Deprescribe, or Stop). To optimize accuracy, clinical notes were preprocessed into summaries before RAG retrieval.

**Results:**

The optimized system achieved an F1 score of 0.84, significantly outperforming the baseline (0.70).

**Conclusion:**

RxReduce demonstrates the viability of artificial intelligence-assisted deprescribing. By extracting key contextual insights, the framework reduces clinician burden and can be adapted for various medication classes to improve patient safety.

## Background and significance

The complexity of medication management in hospitalized patients, particularly during discharge, presents a significant challenge to health systems.^1^ Polypharmacy rates have substantially increased, with rates tripling in patients aged 65 and older between 1998 and 2010.^2^ With 13%-39% of acute geriatric medical admissions being attributed to adverse drug events (ADEs), appropriate discontinuation of unneeded medications could potentially decrease these ADEs.^1^^,^^3^^,^^4^

Deprescribing, defined as the planned process of eliminating medications where the potential for harm outweighs the potential for benefit, is critical for optimizing patient outcomes.^3–5^ Providers often face difficulty and time constraints when performing a patient’s comprehensive medication review as the many of the indications for medications are in patient notes or scattered throughout a patients chart. This creates a time-consuming process for the clinicans.^1^^,^^6^^,^^7^ Often a medication review requires scanning hundreds of pages of clinical notes. The lack of effective supporting tools can result in the continuation of medications past discharge that could potentially be removed.

Papers have demonstrated the capabilities of large language models (LLMs) to parse large corpuses of text for identification of clinically relevant information.^8^ Literature review identified potential applications of technology in the assistance of proper medication deprescribing.^9^^,^^10^ RxReduce was developed to test whether LLMs could be utilized to assist clinicians with the review of medications for the deprescribing process utilizing deidentified electronic medical record (EMR) data. Retrieval-augmented generation (RAG)^11^ is a process where relevant data can be retrieved and inserted into the context window of an LLM. This method enables the selection of data which would exceed the context widow of the model to be identified and utilized by the model. This system was designed to leverage LLMs and RAG to efficiently evaluate EMR patient data and provide referenced recommendations and explanations to assist clinicians in the medication deprescribing process.

## Objective

The purpose of our project was to develop and evaluate RxReduce, an automated medication deprescribing agent framework, specifically designed to assist clinicians by applying defined deprescribing algorithms to complex inpatient data sources.

## Materials and methods

### Target medication class and data sources

This project was completed as part of a capstone by data science master’s program candidates and focused specifically on the deprescribing of proton pump inhibitors (PPIs). They were selected as a proof of concept medication class because they are commonly prescribed and frequently continued in health systems without sufficient supporting indications.^12–14^ All recommendations were based on the PPI deprescribing algorithm from deprescribing.org. A summary of the algorithm can be seen in Figure 1.

**Figure 1.**
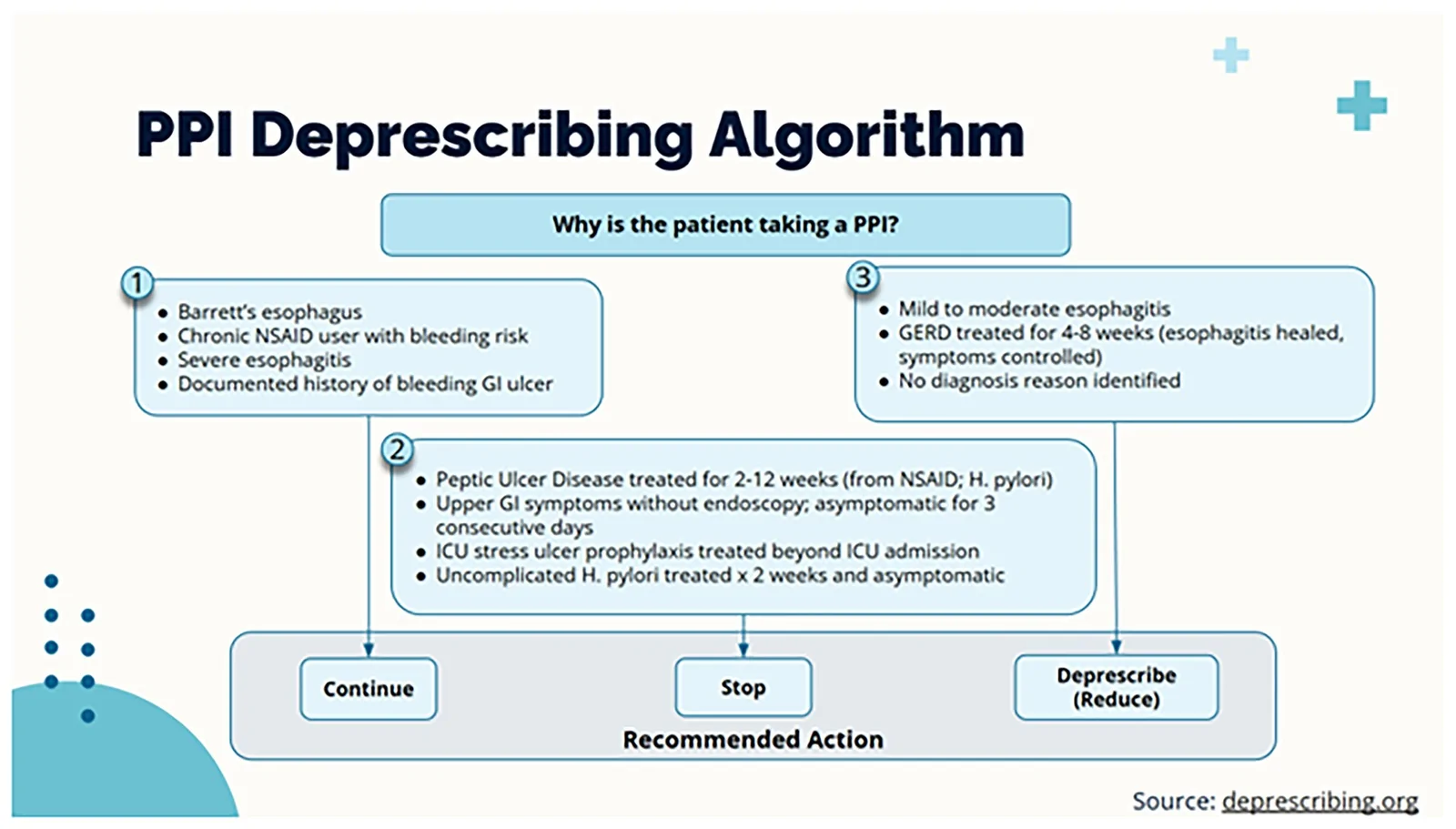
Proton pump inhibitor deprescribing algorithm summary.

The system evaluates 3 primary sources of patient information: patient diagnosis information, inpatient stay information, and clinical notes. Patient data used for development and evaluation consisted of a labeled patient dataset developed from deidentified patient data provided by the University of California, San Francisco (UCSF) Information Commons team. Structured query language code was developed and reviewed prior to pulling sources of data for example patients. As a sample population, inpatient admitted patients were selected based on a hospital discharge date between September 1, 2023, and September 15, 2023 who then continued a PPI at discharge (*n* = 160). Deidentified patient encounter data including primary admission reason, historical diagnosis, stay acquired diagnosis, and clinical notes were pulled from relevant tables. In addition to the de-identified patient data, synthetic patient examples were created based on scenarios encountered within the de-identified data to evaluate the system for specific, complicated scenarios. The group worked to create these hypothetical scenarios based on the deprescribing guidelines which would fit into a path of the deprescribing flow chart. The scenarios were manually written using similar text from the example patient notes. In total, 43 labeled patient examples were considered, including 6 synthetic patients. These patient examples were extracted as static pipe delimited text files which could be used for testing and development. The project team first split and reviewed all patient examples for relevant documentation and labeled each case. A member of the project team who is a registered pharmacist again reviewed all 43 cases and validated the ultimate labeled decision.

### System design and implementation

RxReduce was built as a framework which can integrate with many LLM providers. For development, we utilized the Llama 3.1 and 3.3 70 billion parameter models accessed via the Groq API. Model temperature was set at 0.0 to reduce the possibility of hallucinations.

#### Retrieval and search log

The system processes patient information from various data sources sequentially, assembling a search log that documents evaluation findings and associated metadata. Patient diagnosis and inpatient stay data are included along with filtered patient notes. The filtered patient notes included medication clinically relevant notes from selected provider types (eg, residents, physicians, pharmacists, nurses, etc.). The pre-post removal impact of filtering to appropriate providers can be seen in the Supplementary Material. The distribution of notes and volume of notes was only slightly reduced.

#### Clinical note preprocessing (RAG implementation)

Clinical notes often constitute the largest volume of patient data, potentially numbering in the thousands for patients with a longer length of stay. This correlation with length of stay is visualized in the Supplementary Material. To efficiently process these notes, RAG was utilized as the entirety of notes would exceed the context window of the LLM. The system uses an embedding model trained specifically on medical data.^15^

To improve note evaluation:

1. Each patient note was preprocessed by an LLM to generate a summary of the note.
2. The LLM determined if the note contained information relevant to the medication class in question (PPIs).
3. For inclusion in the vector database used for retrieval, the system found that prepending the LLM-generated summary with the full clinician note text yielded the best performance.

#### Recommendation generation

The system uses the logged evaluation results, which include relevant patient diagnosis information, to apply the PPI deprescribing algorithm and generate a final recommendation. The decision classes considered are as follows: Continue (eg, for Barrett’s esophagus or chronic non-steroidal anti-inflammatory drug (NSAID) use with bleeding risk which is a clear indication to continue), Deprescribe (eg, for uncomplicated *Helicobacter pylori* treatment past 2 weeks but asymptomatic), or Stop (eg, for stress ulcer prophylaxis post-ICU admission). The output includes a written explanation that summarizes the logged findings and cites the relevant patient information that led to the decision.

RxReduce was evaluated in 2 dimensions: as a classifier (predicting the correct recommendation class) and using generative metrics focused on response quality. The classification metrics considered were precision, recall, and F1 score, which was the focus metric.

Generative metrics included RAG assessment (RAGAS) faithfulness and relevancy, BERTScore, METEOR, BLEU, and ROUGE.^16–19^

## Results

Our final system was a minimal viable product (MVP), and we tested multiple configurations. For most patients, the relevant clinical documentation existed in the notes, and as the notes made up the largest portion of the data associated with the patient scenarios, our modifications to the framework centered around this component.

For classification evaluation, 3 system configurations were compared:

1. Baseline MVP: Clinician note texts were considered raw (F1: 0.70).
2. Mini MVP: Only the LLM summaries of the clinician notes were considered (F1: 0.66).
3. Final MVP: The LLM summaries were concatenated with the full clinician note text for RAG retrieval (F1: 0.84).

Figure 2 visualizes the final system configuration process. The system performed best as a classifier when considering the LLM summaries concatenated with the full note text, achieving a Precision of 0.91, Recall of 0.80, and F1 score of 0.84. Comparison results of the 3 configurations can be seen in Table 1. The classification output of the final prepended note summary configuration can be seen in Figure 3. The top left, middle, and bottom right blocks (*n* = 37) of the confusion matrix represented successful classification of the actual to predicted (continue–continue, deprescribe–deprescribed, or stop–stop). The remaining blocks (*n* = 6) demonstrated mismatches between the labeled and predicted.

**Figure 2.**
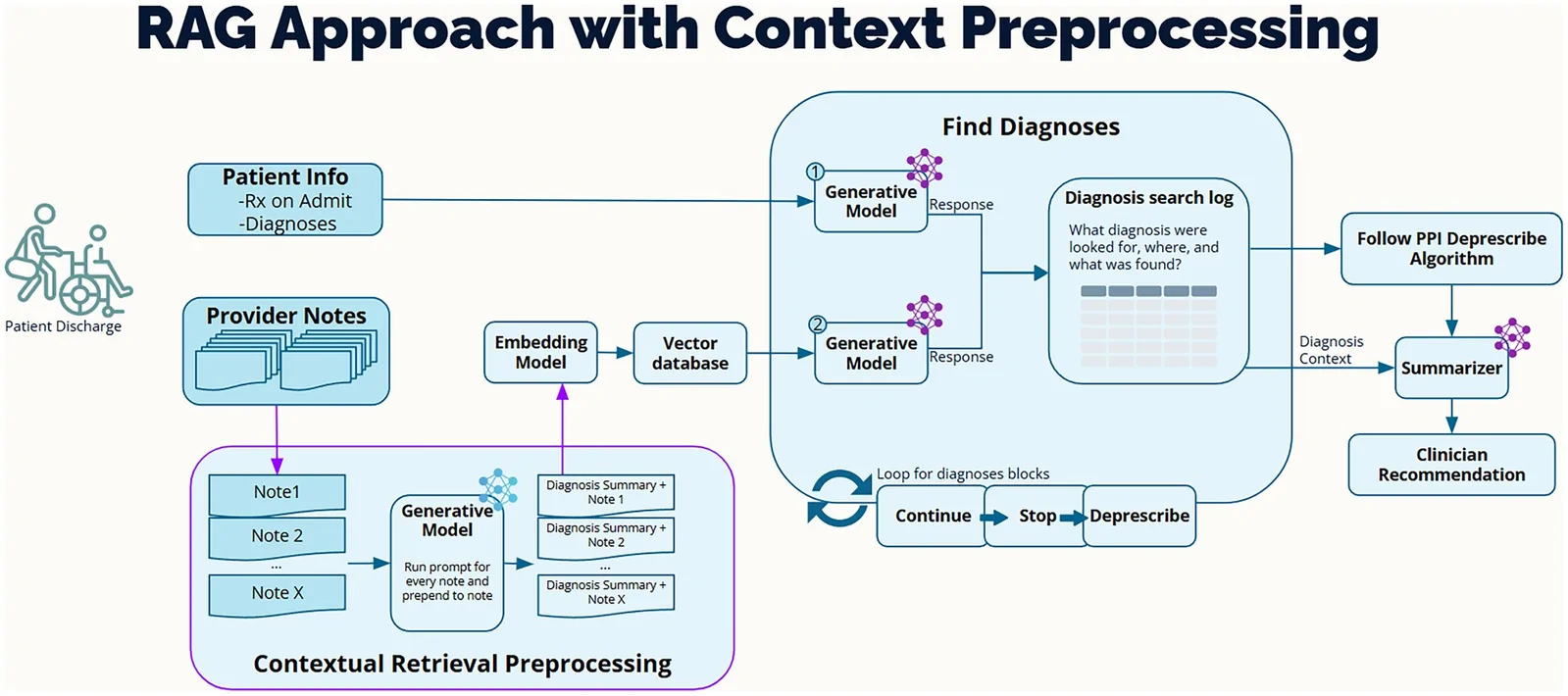
Final system configuration with preprocessed summary prepending.

**Figure 3.**
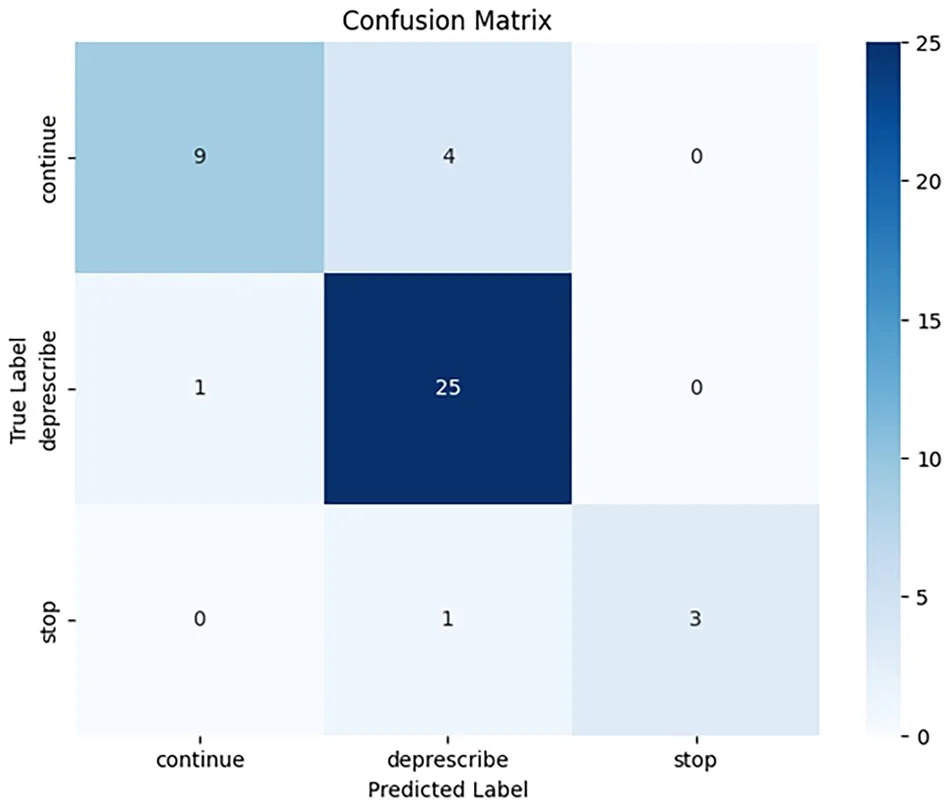
Confusion matrix for final system recommendation classification.

**Table 1.** Notes method classification results.

Model	Notes retrieval	Precision	Recall	F1
Baseline MVP	Full note text	0.86	0.64	0.70
Mini MVP	Preprocessed summary only	0.68	0.67	0.66
Final MVP	Preprocessed summary + full note text	**0.91**	**0.80**	**0.84**

Abbreviation: MVP, minimal viable product.  Bold text indicates the top performing metric for each note retrieval method.

Table 2 presents the evaluation metrics for the 3 retrieval methods, with bold text indicating the top-performing score within each metric.

**Table 2.** Notes method retrieval results.

Dataset	BERT precision	BERT recall	BERT F1	Rouge F1	METEOR	BLEU
Baseline MVP	**0.845**	0.826	0.8349	0.192	0.148	0.0044
Mini MVP	0.843	0.827	0.8346	0.194	0.142	0.0042
Final MVP	0.834	**0.839**	**0.8363**	**0.202**	**0.157**	**0.0048**

Abbreviation: MVP, minimal viable product.  Bold text indicates the top performing metric for each note retrieval method.

While semantic similarity scores via BERTScore remained consistently high across all models, the final MVP achieved the highest BERT F1 (0.8363) and BERT recall (0.8390). In the context of medical text processing, a BERT F1 slightly below 0.85 is typical and acceptable here, as the task involves synthesizing highly variable clinical notes rather than extracting rigid, verbatim terminology.^20–22^

For the n-gram surface-level metrics, the final MVP consistently outperformed the Baseline and Mini variants, securing the highest Rouge F1 (0.202), METEOR (0.157), and BLEU (0.0048) scores. Though these absolute values appear low (eg, Rouge and METEOR below 0.5; BLEU below 0.01), they do not indicate poor model performance. Traditional n-gram overlap metrics are notoriously penalized by the inherent syntactic flexibility, diverse vocabulary, and varied phrasing characteristic of medical note summarizing and retrieval.^20–22^ Because there are many valid ways to structure clinical narratives, a low direct word-for-word overlap is expected and does not reflect a deficiency in factual or clinical utility, as corroborated by the robust, alignment-insensitive BERTScore metrics.

Manual analysis of synthetic patient cases demonstrated the system’s ability to extract key contextual information. As an example, for Synthetic Patient 1 (Labeled Recommendation: Stop), the system correctly recommended stop based on notes indicating the patient had completed the 14-day treatment course for *H. pylori* infection and was scheduled for follow-up testing. However, the ambiguity in clinical decision-making was also evident as the system often provided reasonable alternative recommendations given the patient data, highlighting the difficulty and time commitment of accurate deprescribing faced by clinicians. Token consumption varied between deprescribing scenarios due to the length of the patient notes and stopping point during the deprescribing evaluation. Average token consumption per scenario was roughly 20 000 tokens.

## Discussion

This project demonstrated the viability of an LLM-powered solution in assisting providers with appropriate medication deprescribing recommendations. The ability of RxReduce to identify and flag relevant note sections and diagnosis information is valuable, especially for patients with high note volumes which we found correlated with inpatient stay length. Studies have demonstrated that patient increased length of stay is correlated with polypharmacy and successfully deprescribing medications to reduce the prevalence of polypharmacy could also have an impact on length of stay.^23^ Clinicians consulted confirmed the difficulties and time commitment of the discharge medication process and the need for improved technology.

The primary technical finding was the increased accuracy achieved by distributing the search into a sequence to build a system search log, which ensures relevant contextual information is referenced along with the prepending summary technique described which increased the accuracy of the classification. While the current evaluation focused on single recommendations for classification purposes, future deployment will encourage the output of multiple recommendations with supporting evidence, providing the clinician with more complete information and greater decision-making power. The overall framework is flexible and can be expanded to apply to additional medication classes beyond PPIs and adopted to institution specific or national deprescribing guidelines.

The risk of misclassification with a deprescribing system can manifest as 2 scenarios. If a medication is incorrectly classified as flagged for deprescribing when it should be continued, this can result in under treatment of the patient condition and lead to disease progression. Alternatively, medications which could be discontinued and are flagged for continuation could lead to increased rates of polypharmacy, side effects, medication interactions, and cost for the patient and/or health system. With these failure scenarios in mind, our system was intentionally designed as a “provider in the loop” decision augmentation solution.

### Limitations

The evaluation of this framework was limited by the number of labeled cases in the test data set. Ideally, multiple providers would have reviewed each patient deprescribing scenario and a larger number of scenarios would have been included. Due to time and resource limitations, we were unable to include a larger number of scenarios. The lack of availability of labeled data sets and time requirement of creating our own, speaks to the complexity of deprescribing and properly evaluating the appropriate decision for a patient’s medications.

## Conclusion

RxReduce is a framework that leverages AI techniques (LLMs and RAG) to assist clinicians with the challenges associated with complex medication deprescribing. By automating the extraction and synthesis of relevant patient information according to an established deprescribing algorithm, the system demonstrates the potential to reduce the cognitive burden and time commitment required for providers. Integration of deprescribing clinical decision tools into EMRs can assist providers with optimal medication use and have the potential to reduce the time barrier present with effective deprescribing. All code and evaluations can be accessed via the GitHub link provided.

## Supplementary Material

ooag167_Supplementary_Data

## Data Availability

The data underlying this article cannot be shared publicly due to the privacy considerations associated with the deidentified patient data provided by the University of California, San Francisco (UCSF) Information Commons team. However, synthetic patient data developed for specific scenarios may be shared on reasonable requests to the corresponding author. Code is available at https://github.com/Beenjamming/DataSci210_MedicationDeprescriber

## References

[1] Michaelsen M , McCague P, Bradley C, Sahm L. Medication reconciliation at discharge from hospital: a systematic review of the quantitative literature. Pharmacy. 2015;3:53-71. 10.3390/pharmacy3020053 PMC5597088 28975903

[2] Charlesworth CJ , Smit E, Lee DSH, Alramadhan F, Odden MC. Polypharmacy among adults aged 65 years and older in the United States: 1988–2010. J Gerontol A: Biol Sci Med Sci. 2015;70:989-995. 10.1093/gerona/glv013 PMC4573668 25733718

[3] Linsky AM , Motala A, Lawson E, Shekelle P. Deprescribing to *Reduce Medication Harms* in *Older Adults: A Rapid Response*. Washington, DC: Department of Veterans Affairs (US); 2023. VA Evidence Synthesis Program Reports. https://www.ncbi.nlm.nih.gov/books/NBK600387/38349984

[4] Thompson W , Farrell B. Deprescribing: what is it and what does the evidence tell us? Can J Hosp Pharm. 2013;66:201-202. 10.4212/cjhp.v66i3.1261 PMC3694945 23814291

[5] Scott IA , Hilmer SN, Reeve E, et al Reducing inappropriate polypharmacy: the process of deprescribing. JAMA Intern Med. 2015;175:827-834. 10.1001/jamainternmed.2015.0324 25798731

[6] Robinson M , Mokrzecki S, Mallett AJ. Attitudes and barriers towards deprescribing in older patients experiencing polypharmacy: a narrative review. NPJ Aging. 2024;10:6. 10.1038/s41514-023-00132-2 PMC10806180 38263176

[7] Zidan A , Awaisu A. Inappropriate polypharmacy management versus deprescribing: a review on their relationship. Basic Clin Pharmacol Toxicol. 2024;134:6-14. 10.1111/bcpt.13920 37350370

[8] Akyon SH , Akyon FC, Yılmaz TE. Artificial intelligence-supported web application design and development for reducing polypharmacy side effects and supporting rational drug use in geriatric patients. Front Med (Lausanne). 2023;10:1029198. 10.3389/fmed.2023.1029198 PMC10030839 36968816

[9] Pourian JJ , Michaels B, Vo A, Holmgren AJ, Garcia-Agundez A, Flaherman V. A SNAPpy use of large language models: using large language models to classify treatment plans in pediatric acute otitis media. J Am Med Inform Assoc. 2025;32:1947-1951. 10.1093/jamia/ocaf170 PMC12646383 41069025

[10] Rao A , Kim J, Lie W, et al Proactive polypharmacy management using large language models: opportunities to enhance geriatric care. J Med Syst. 2024;48:41. 10.1007/s10916-024-02058-y PMC12042286 38632172

[11] Lewis P , Perez E, Piktus A, et al Retrieval-augmented generation for knowledge-intensive NLP Tasks. *Adv Neural Inf Process Syst*. 2020;33. http://arxiv.org/abs/2005.11401.

[12] Rossi A , Perrella L, Scotti S, et al Approaches to deprescribing proton pump inhibitors in clinical practice: a systematic review. J Clin Med. 2024;13:1-20. 10.3390/jcm13206283 PMC11508458 39458232

[13] Farrell B , Pottie K, Thompson W, et al Deprescribing proton pump inhibitors: Evidence-based clinical practice guideline. Can Fam Physician. 2017;63:354-364. PMC5429051 28500192

[14] Andrawes M , Andrawes W, Das A, Siau K. Proton pump inhibitors (PPIs)—an evidence-based review of indications, efficacy, harms, and deprescribing. Medicina (Lithuania). 2025;61:1569. 10.3390/medicina61091569 PMC12471631 41010960

[15] Gu Y , Tinn R, Cheng H, et al Domain-specific language model pretraining for biomedical natural language processing. ACM Trans Comput Healthc. 2022;3:1-23. 10.1145/3458754

[16] Ghassemiazghandi M. An evaluation of ChatGPT’s translation accuracy using BLEU score. Theory Pract Lang Stud. 2024;14:985-994. 10.17507/tpls.1404.07

[17] Akhmetov I , Mussabayev R, Gelbukh A. Reaching for upper bound ROUGE score of extractive summarization methods. PeerJ Comput Sci. 2022;8:e1103. 10.7717/peerj-cs.1103 PMC9575858 36262160

[18] Ahmed R , Hemanth DJ. Hybrid text summarization: Integrating extractive and abstractive models for enhanced cross-domain summarization. Intell. Decision Technologies. 2025;19:2664-2679. 10.1177/18724981251322745

[19] Zhang T , Kishore V, Wu F, Weinberger KQ, Artzi Y. BERTScore: evaluating text generation with Bert. In: *8th International Conference on Learning Representations*. ICLR; 2020.

[20] Junadhi J , Agustin A, Efrizoni L, Okmayura F, Habibie DR, Muslim M. Improving evaluation metrics for text summarization: a comparative study and proposal of a novel metric. J Appl Data Sci. 2025;6:885-896. 10.47738/jads.v6i2.547

[21] Vasilev Y , Raznitsyna I, Arzamasov K, et al Evaluating medical text summaries using automatic evaluation metrics and LLM-as-a-judge approach: a pilot study. Diagnostics. 2025;15:12786185. 10.41515/j.diagnostics.2025.547 PMC12786185 41515497

[22] Tang L , Sun Z, Idnay B, et al Evaluating large language models on medical evidence summarization. *npj Digit. Med*. 2023;6:158. 10.1038/s41746-023-00896-7 PMC10449915 37620423

[23] Fukuba N , Nishida M, Hayashi M, et al The relationship between polypharmacy and hospital-stay duration: a retrospective study. Cureus. 2020;12:e7267. 10.7759/cureus.7267 PMC7153814 32292678

